# Causal Association Between 12 Micronutrients and Common Chronic Respiratory Diseases: A Bidirectional Two-Sample Mendelian Randomization Study

**DOI:** 10.1155/genr/7575005

**Published:** 2025-05-28

**Authors:** Tingting Zhu, Xiuyun Chen, Qing Wang, Fang Li, Junjun Yang, Xinyu Zhu, Jingmei Wang, Jixiang Bo

**Affiliations:** Department of Respiratory and Critical Care Medicine, Northern Jiangsu People's Hospital, Yangzhou 225000, Jiangsu, China

**Keywords:** causal relationship, chronic respiratory diseases, Mendelian randomization, micronutrients

## Abstract

**Background:** This study aims to investigate the causal relationships between 12 micronutrients and common chronic respiratory diseases, revealing whether these nutrients play a causative role in either preventing or exacerbating these conditions.

**Methods:** We employed a bidirectional two-sample Mendelian randomization (MR) approach to explore the causal relationships between micronutrients and chronic respiratory diseases. Data were sourced from the IEU GWAS database, with micronutrients serving as exposure variables and chronic respiratory diseases as outcome variables for causal assessment. This was followed by reverse MR analysis, where the steps were reversed. Analytical methods included inverse-variance weighting (IVW), MR-Egger regression, and the weighted median method to correct for potential pleiotropy and reverse causality. Cochran's *Q* test and the MR-PRESSO method were used for pleiotropy tests to ensure robustness and reliability of the results.

**Results:** The MR analysis revealed that the genetically predicted calcium is a protective factor for asthma (OR = 0.99, 95% CI 0.984–0.995, *p* < 0.01), vitamin B12 is a risk factor for asthma (OR = 1.015, 95% CI 1.005–1.024, *p* < 0.01), and vitamin E is a protective factor for idiopathic pulmonary fibrosis (IPF) (OR = 0.952, 95% CI 0.916–0.989, *p*=0.012). In the reverse MR analysis, asthma showed a potential causal relationship with calcium levels (OR = 0.829, 95% CI 0.704–0.976, *p*=0.025), while pneumoconiosis showed a potential risk causal relationship with calcium levels (OR = 1.003, 95% CI 1.002–1.004, *p* < 0.010). Additionally, pneumoconiosis was found to have a potential protective causal relationship with vitamin E levels (OR = 0.999, 95% CI 0.999–1.000, *p*=0.034), and sarcoidosis was found to have a potential protective causal relationship with vitamin B12 levels (OR = 0.989, 95% CI 0.979–1.000, *p*=0.044).

**Conclusion:** This study shows significant causal associations among calcium, vitamin B12, and vitamin E with chronic respiratory diseases. There is a bidirectional protective causal relationship between calcium and asthma, suggesting that increasing calcium intake may reduce the risk of asthma. However, the causal relationships among other vitamins, minerals, and chronic respiratory diseases remain inconclusive, necessitating further research to validate these findings' robustness and generalizability.

## 1. Introduction

Chronic respiratory diseases (CRDs) are significant global causes of morbidity and mortality, encompassing conditions such as chronic obstructive pulmonary disease (COPD), asthma, idiopathic pulmonary fibrosis (IPF), sarcoidosis, and pneumoconiosis [1]. According to the World Health Organization (WHO), COPD is the fourth leading cause of death worldwide, resulting in over 3 million deaths annually. Asthma affects more than 300 million people globally, leading to substantial social and economic burdens [2]. IPF is a fatal lung disease with an average survival time of only 3–5 years [3]. Sarcoidosis is a multisystem inflammatory disease with an incidence rate of 10–20 per 100,000 people [4]. Pneumoconiosis is a major occupational disease, severely impacting the health of miners and other high-risk occupational groups [5]. These diseases not only severely affect patients' quality of life but also place a tremendous burden on global healthcare systems.

Vitamins and minerals are essential micronutrients and inorganic nutrients required by the human body. Despite their relatively low levels in the body, they play indispensable roles in maintaining the normal functioning of life processes. In recent years, increasing research has revealed the complex roles of vitamins and minerals in disease pathogenesis. Vitamins A, D, E, and K are fat-soluble vitamins with significant roles in cell signaling and gene regulation [6]. Vitamin D, through its receptor (VDR) in immune cells, regulates innate and adaptive immune responses, playing a crucial role in combating infections and inflammation [7]. Vitamin E, as a potent antioxidant, can neutralize free radicals and reduce oxidative stress-induced cellular damage, thereby contributing to the prevention and treatment of cardiovascular diseases, neurodegenerative diseases, and other conditions [8, 9]. Water-soluble vitamins, such as vitamin C and the B vitamins, also exhibit significant biological functions in various diseases. Vitamin C enhances immune function and aids in wound healing and infection resistance by promoting collagen synthesis [10]. B vitamins are crucial for energy metabolism, nervous system health, and DNA synthesis and repair, with deficiencies often associated with anemia, neurological damage, and cardiovascular diseases [11].

Macrominerals such as calcium, magnesium, potassium, and sodium are present in higher amounts in the body and are vital for maintaining bone health, nerve conduction, muscle contraction, and heart function. Calcium is not only a major component of bones and teeth but also plays roles in blood coagulation, nerve signal transmission, and regulation of intracellular and extracellular signals [12]. Magnesium is key in energy metabolism, protein synthesis, and nucleic acid stability, with deficiencies linked to cardiovascular diseases, metabolic syndrome, and neurological disorders [13]. Trace minerals like iron, zinc, copper, and selenium, although present in minute amounts, are equally critical for physiological functions. Iron is a core component of hemoglobin, responsible for oxygen transport, with deficiencies leading to anemia and fatigue [14]. Zinc is essential for cell division, immune function, and enzyme activity, with deficiencies associated with impaired growth, weakened immunity, and increased infection risk [15]. Selenium, as an important antioxidant, protects cells from oxidative stress damage and plays a role in thyroid function regulation [16].

Although observational studies have suggested associations between micronutrients and CRDs—for instance, vitamin D deficiency has been linked to worsened asthma, and zinc deficiency may increase the infection risk in COPD patients—these associations do not establish causality. Observational studies are often limited by confounding factors and reverse causality, making it difficult to draw definitive causal conclusions. To address these issues, the Mendelian randomization (MR) method has emerged. MR uses genetic variations as instrumental variables, analyzing the relationships between genes and exposure and outcomes, thus overcoming the limitations of observational studies [17]. This method is based on the principle of random allocation of genetic variations at conception, making it similar to a natural randomized controlled trial, thereby providing stronger causal inference [18]. Bidirectional two-sample MR further enhances causal inference capabilities by using associations among genetic variations, exposure, and outcomes from two independent samples, reducing bias from reverse causality.

This study aims to use bidirectional two-sample MR to explore the causal relationships between 12 micronutrients and common CRDs. The goal is to reveal whether these micronutrients play causative roles in either preventing or exacerbating CRDs, thereby providing new scientific evidence for disease prevention and treatment.

## 2. Materials and Methods

### 2.1. Study Design

**Figure 1:**
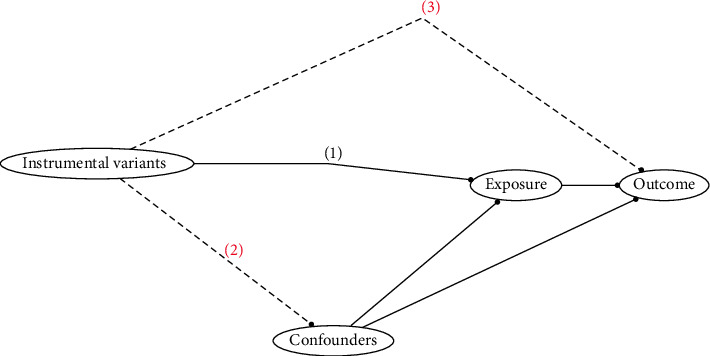
The instrumental variable assumptions in Mendelian randomization studies. (1) Relevance assumption: SNP is strongly associated with the exposure factor; (2) independence assumption: SNP is independent of confounding factors; and (3) exclusion restriction assumption: SNP affects the outcome only through the exposure factor.

This study employed a bidirectional two-sample MR approach to explore the causal relationships between 12 micronutrients and common CRDs. MR is a method that uses genetic variations as instrumental variables to assess causal relationships between exposures and outcomes. The core idea of MR is to use genetic variations (SNPs) associated with exposures as instrumental variables. These must satisfy three assumptions to ensure the validity of the instrumental variables as proxies for the exposures, thus allowing the gene-exposure associations to infer causality between the exposures and outcomes (Figure 1). Bidirectional two-sample MR further enhances the reliability of causal inference. Firstly, a traditional unidirectional two-sample MR analysis will be conducted. This involves using data on the associations between genetic variations and CRDs (exposures) as the first sample and data on the associations between genetic variations and micronutrient levels (outcomes) as the second sample. By analyzing these data, we infer the causal effects of micronutrient levels on CRDs. To rule out the possibility of reverse causality, a reverse analysis will be conducted, which is the reverse of the first step.

### 2.2. Data Sources

The data for this study were sourced from the IEU GWAS database (https://gwas.mrcieu.ac.uk/). This database provides detailed association data among common CRDs, micronutrient levels, and genetic variations, identifying and including SNPs significantly associated with both common CRDs and micronutrient levels. Specifically, we collected data on calcium, copper, iron, magnesium, selenium, zinc, vitamin B12, vitamin B6, vitamin C, vitamin A, vitamin D, and vitamin E (Table 1). These datasets encompass diverse populations and large sample sizes, ensuring the broad applicability and reliability of the study results.

For common CRDs, this study included the following sample sizes: COPD (468,475 samples, 24,180,654 SNPs), asthma (484,598 samples, 9,587,836 SNPs), IPF (451,025 samples, 16,137,102 SNPs), sarcoidosis (486,673 samples, 24,196,796 SNPs), and pneumoconiosis (479,040 samples, 24,200,183 SNPs).

### 2.3. Instrumental Variable Selection Criteria

First, we will select SNPs significantly associated with each micronutrient as instrumental variables [19]. These genetic variations need to meet the criteria for strong instrumental variables, meaning their association with the micronutrient is statistically significant, typically with a *p* value less than 5*e* − 08. Selecting these genetic variations ensures that their impact on the exposure variables is strong enough to provide reliable causal inference. If there are not enough instrumental variables at this threshold, the threshold will be gradually lowered, with a minimum *p* value of 5*e* − 06, to ensure a sufficient number of instrumental variables for the analysis. Additionally, SNPs in linkage disequilibrium (LD) need to be filtered out to ensure the independence of the selected instrumental variables. The specific screening criterion is an LD coefficient (*r*^2^ < 0.001). This ensures the independence of the instrumental variables, avoiding multicollinearity issues due to correlations among genetic variations. To further validate the strength of the instrumental variables, we calculate the *F*-statistic for each set of MR analyses, with an *F*-value greater than 10 indicating a strong instrumental variable. The *F*-value measures the variance explained by instrumental variables to exposure variables, and the *F*-values of all results are greater than 10, indicating that there is sufficient explanatory power to provide reliable causal effect estimates.

### 2.4. MR Analysis

To understand the causal effects between micronutrients and CRDs, multiple MR analysis methods will be employed using the TwoSampleMR package (version 0.5.7) in R. First, the inverse-variance weighted (IVW) method is the primary analysis method used to estimate the overall causal effect of micronutrients on diseases. The IVW method improves estimation accuracy by averaging the effects of multiple genetic variations, weighted by their precision. The basic idea is to perform a weighted regression of each SNP's association with the exposure and outcome, thereby estimating the overall effect. Second, MR-Egger regression is used to detect and correct for potential pleiotropy of genetic variations, meaning the genetic variations may influence the outcome variable through multiple pathways, not just the micronutrient. MR-Egger regression provides causal effect estimates corrected for pleiotropic effects. Finally, when there are partially invalid instrumental variables, the weighted median method provides robust causal effect estimates [20].

Additionally, to further validate the analysis results, we will use the leave-one-out method, gradually removing each instrumental variable and calculating the meta-effect of the remaining instrumental variables to observe if the results change significantly when any single instrumental variable is removed. If removing a specific instrumental variable significantly alters the results, that variable will be excluded. When sensitivity analyses indicate pleiotropy, the MR-PRESSO method will be used to detect and exclude outliers, and the analysis will be repeated. If pleiotropy persists, the analysis will be excluded. To ensure the reliability and robustness of the analysis results, the main MR analysis results will primarily rely on the IVW method. Positive results are determined if the beta values of IVW, weighted median, and MR-Egger methods are consistent in direction (all positive or all negative), and at least the IVW method has a *p* value less than 0.05.

### 2.5. Robustness Tests and Sensitivity Analysis

To validate the robustness of the study results, a series of robustness tests and sensitivity analyses will be performed [21]. First, Cochran's *Q* statistic and the *I*^2^ statistic will be used to detect heterogeneity among the effects of different genetic variations. Significant heterogeneity indicates differences in the effects of different SNPs, necessitating further exploration of the causes and adjustment of the analysis model. A *Q*_*p*val greater than 0.05 indicates no significant heterogeneity. In the presence of heterogeneity, results from the weighted median or IVW random-effects model will be used; in the absence of heterogeneity, the IVW fixed-effects model will be used. Second, the intercept term of the MR-Egger regression will be used to assess the presence of pleiotropy. A significant nonzero intercept indicates pleiotropy, necessitating correction for pleiotropy to obtain accurate causal effect estimates. The MR-PRESSO method will be used to further test for and exclude pleiotropic outliers. Finally, sensitivity analysis will verify the robustness of the study results by excluding potential outliers or invalid instrumental variables. This includes re-analysis after excluding SNPs with exceptionally large effects or those identified as pleiotropic, observing whether the results change significantly. If the results remain consistent across these sensitivity analyses, we can have greater confidence in the robustness of the causal effects.

## 3. Results

### 3.1. Causal Relationship Analysis of SNPs Related to CRDs

**Figure 2:**
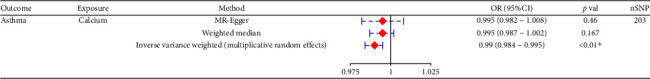
Mendelian randomization analysis of minerals with common chronic respiratory diseases, illustrating the causal relationship between calcium levels and asthma risk. Analytical methods include MR-Egger, weighted median, and inverse-variance weighted (IVW) methods, using 260 SNPs. Each red diamond represents the OR estimate, with *p* values indicating statistical significance. ^∗^*p* < 0.05.

**Figure 3:**
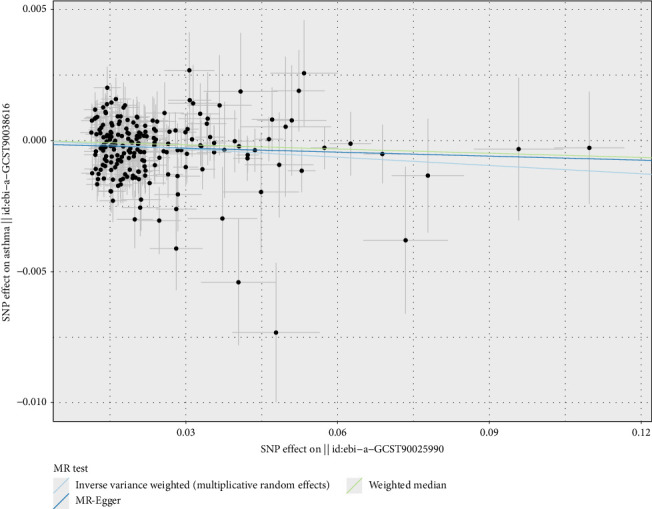
Two-sample Mendelian randomization scatter plot showing the impact of calcium levels on asthma risk. The scatter points represent the SNP effect estimates and their 95% confidence intervals. The horizontal axis shows the effect of SNPs on calcium levels, while the vertical axis shows the effect of SNPs on asthma risk.

In the MR analysis using micronutrients as exposures and CRDs as outcomes, positive results were primarily derived using the IVW method, with consistent trends observed in both MR Egger and weighted median methods. This study found causal relationships between calcium, vitamin B12, vitamin E, and CRDs. For minerals, calcium was identified as a potential protective factor for asthma (OR = 0.99, 95% CI 0.984–0.995, *p* < 0.01) (Figure 2). A total of 203 SNPs were included, all of which had ORs less than 1, indicating that each standard deviation (SD) increase in calcium levels was associated with a 1.00% reduction in the probability of developing asthma (Figure 3).

**Figure 4:**
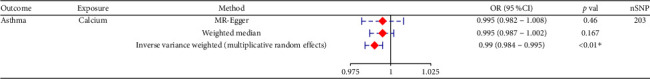
Mendelian randomization analysis of vitamins with common chronic respiratory diseases, illustrating the causal relationships between vitamin B12 levels and asthma risk, and vitamin E levels and idiopathic pulmonary fibrosis (IPF) risk. Analytical methods include MR-Egger, weighted median, and inverse-variance weighted (IVW) methods, using 9 and 14 SNPs. Each red diamond represents the OR estimate, with *p* values indicating statistical significance. ^∗^*p* < 0.05.

**Figure 5:**
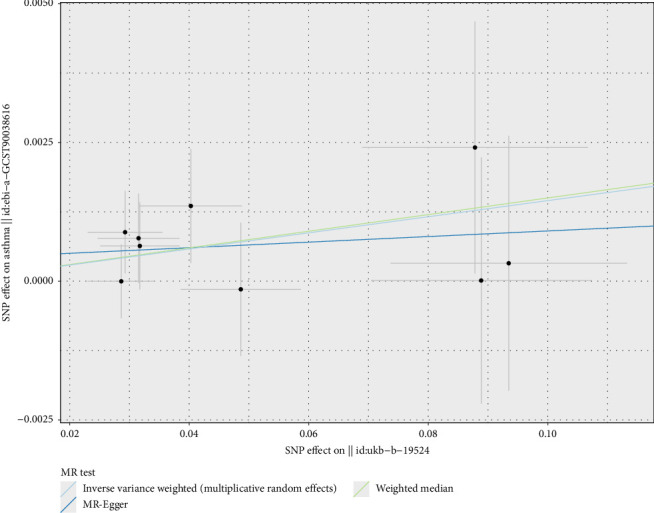
Two-sample Mendelian randomization scatter plot showing the impact of vitamin B12 levels on asthma risk. The scatter points represent the SNP effect estimates and their 95% confidence intervals. The horizontal axis shows the effect of SNPs on vitamin B12 levels, while the vertical axis shows the effect of SNPs on asthma risk.

**Figure 6:**
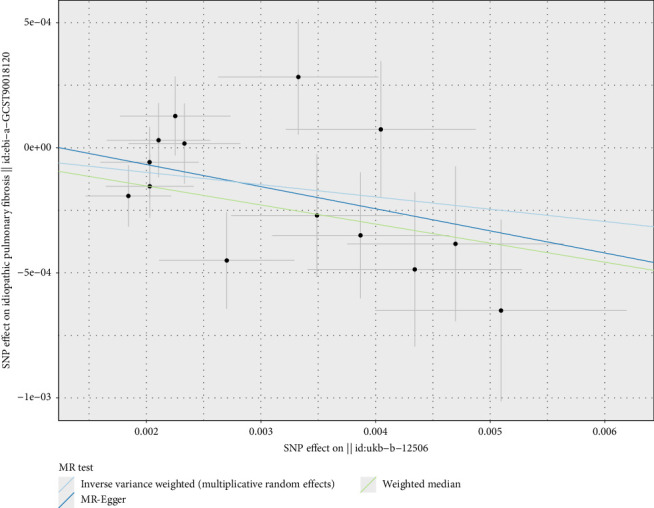
Two-sample Mendelian randomization scatter plot showing the causal relationship between vitamin D levels and idiopathic pulmonary fibrosis (IPF) risk. The scatter points represent the SNP effect estimates and their 95% confidence intervals. The horizontal axis shows the effect of SNPs on vitamin D levels, while the vertical axis shows the effect of SNPs on IPF risk.

For vitamins, the analysis indicated that vitamin B12 might be a potential risk factor for asthma (OR = 1.015, 95% CI 1.005–1.024, *p* < 0.01) (Figures 4 and 5), with ORs greater than 1, suggesting that each SD increase in vitamin B12 levels was associated with a 1.015-fold increase in the probability of developing asthma. Vitamin E, on the other hand, was identified as a potential protective factor for IPF (OR = 0.952, 95% CI 0.916–0.989, *p*=0.012) (Figures 4 and 6), with ORs less than 1, indicating that each SD increase in vitamin E levels was associated with a 4.80% reduction in the probability of developing IPF. These results were consistent and robust across multiple methods. The significance of calcium, vitamin B12, and vitamin E in respiratory health warrants further confirmation, providing valuable insights for potential dietary and therapeutic interventions.

To assess the heterogeneity among SNPs, we performed Cochran's *Q* test for each exposure-outcome pair (Supporting Tables 1 and 2). Significant heterogeneity was observed for calcium in IPF (*Q* = 17.8438, *p*=0.0013, *I*^2^ = 71.98%) and for selenium in pneumoconiosis (*Q* = 4.5998, *p*=0.032, *I*^2^ = 56.52%), suggesting variability in the SNP effects. In cases of significant heterogeneity, we used random-effects models to account for the variation among SNPs. The application of random-effects models allowed for more reliable estimates of causal relationships despite the observed heterogeneity. Similar heterogeneity was observed for other micronutrients, which were addressed using the same approach.

### 3.2. Causal Relationship Analysis of Micronutrient-Related SNPs

**Figure 7:**
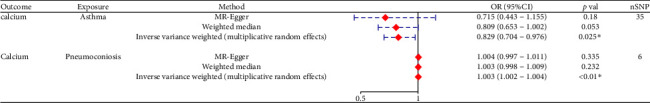
Mendelian randomization analysis of chronic respiratory disease risk with minerals, showing the causal relationships between asthma and pneumoconiosis with calcium levels. Analytical methods include MR-Egger, weighted median, and inverse-variance weighted (IVW) methods, using 35 and 6 SNPs. Each red diamond represents the OR estimate, with *p* values indicating statistical significance. ^∗^*p* < 0.05.

**Figure 8:**
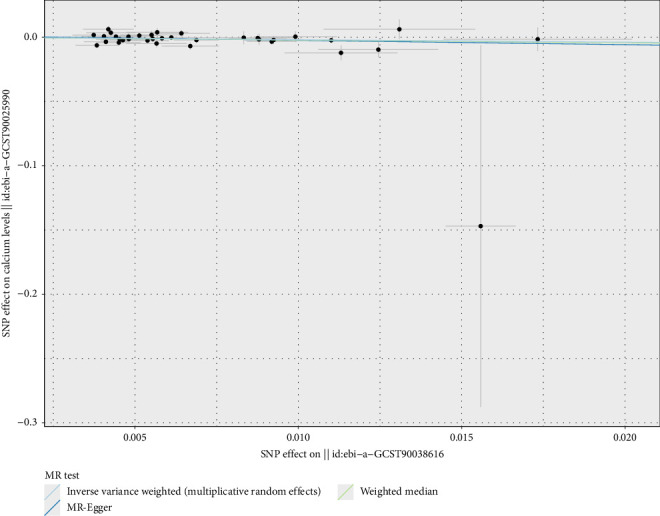
Two-sample Mendelian randomization scatter plot showing the impact of asthma on calcium levels. The scatter points represent the SNP effect estimates and their 95% confidence intervals. The horizontal axis shows the effect of SNPs on asthma, while the vertical axis shows the effect of SNPs on calcium levels.

**Figure 9:**
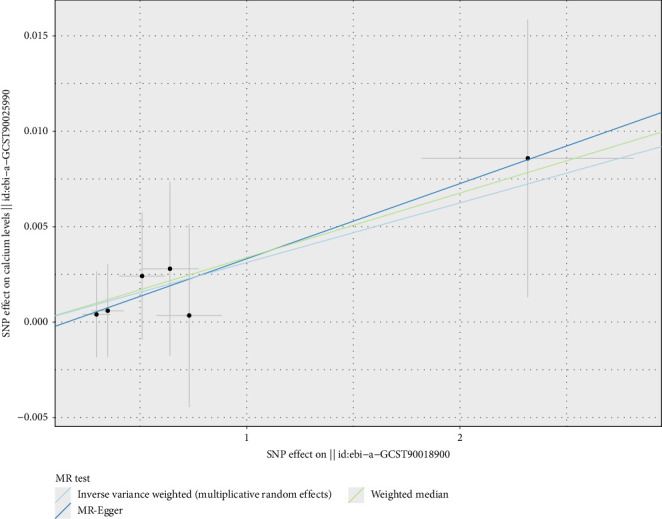
Two-sample Mendelian randomization scatter plot showing the impact of pneumoconiosis on calcium levels. The scatter points represent the SNP effect estimates and their 95% confidence intervals. The horizontal axis shows the effect of SNPs on pneumoconiosis, while the vertical axis shows the effect of SNPs on calcium levels.

Secondly, by conducting reverse MR analysis where CRDs are considered exposures and micronutrients as outcomes, we investigated the potential causal relationships between CRDs and specific micronutrient levels. The results indicated that asthma (OR = 0.829, 95% CI 0.704–0.976, *p*=0.025) potentially has a causal relationship with calcium levels (Figures 7 and 8), with an OR less than 1, suggesting that asthma might exert a protective effect by reducing calcium levels. On the other hand, pneumoconiosis showed a potential risk causal relationship with calcium levels (OR = 1.003, 95% CI 1.002–1.004, *p* < 0.010) (Figures 7 and 9). Statistically speaking, this result is statistically significant, indicating that the possibility of these associations caused by random errors is very low. It shows that pneumoconiosis may increase the risk by increasing the blood calcium level, but because its risk change is far below the threshold that needs to be considered in clinical decision-making, and to achieve meaningful group prevention effect, it is necessary to intervene a very large population to confirm the clinical value, and the conclusion is only of statistical value.

**Figure 10:**
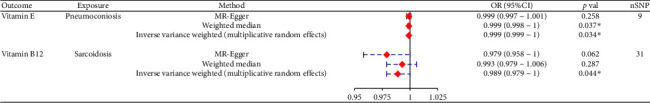
Mendelian randomization analysis of chronic respiratory disease risk with vitamins, showing the causal relationships between pneumoconiosis and vitamin E levels, and sarcoidosis risk and vitamin B12 levels. Analytical methods include MR-Egger, weighted median, and inverse-variance weighted (IVW) methods, using 9 and 31 SNPs. Each red diamond represents the OR estimate, with *p* values indicating statistical significance. ^∗^*p* < 0.05.

**Figure 11:**
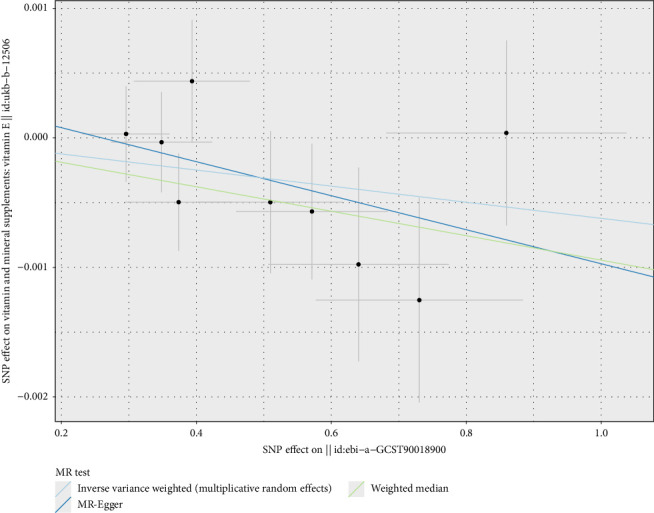
Two-sample Mendelian randomization scatter plot showing the impact of pneumoconiosis on vitamin E levels. The scatter points represent the SNP effect estimates and their 95% confidence intervals. The horizontal axis shows the effect of SNPs on pneumoconiosis, while the vertical axis shows the effect of SNPs on vitamin E levels.

**Figure 12:**
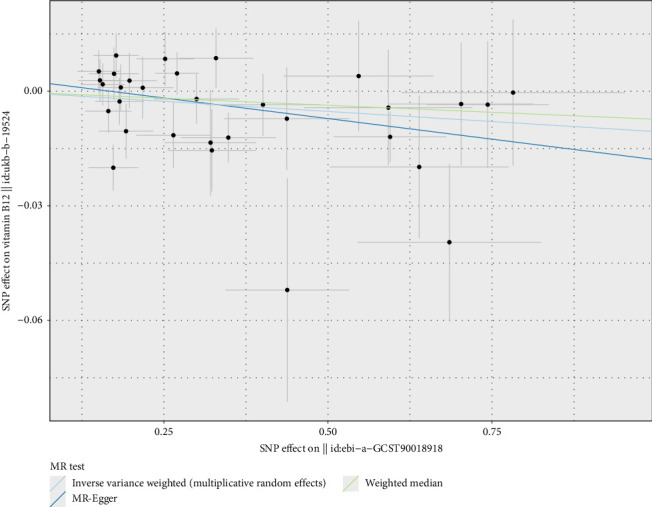
Two-sample Mendelian randomization scatter plot showing the impact of sarcoidosis on vitamin B12 levels. The scatter points represent the SNP effect estimates and their 95% confidence intervals. The horizontal axis shows the effect of SNPs on sarcoidosis, while the vertical axis shows the effect of SNPs on vitamin B12 levels.

Regarding vitamins E, pneumoconiosis exhibited a potential causal relationship with vitamin E levels (OR = 0.999, 95% CI 0.999–1.000, *p*=0.034) (Figures 10 and 11), with an OR less than 1, indicating that pneumoconiosis might exert a protective effect by reducing vitamin E levels. Similarly, sarcoidosis demonstrated a potential causal relationship with vitamin B12 levels (OR = 0.989, 95% CI 0.979–1.000, *p*=0.044) (Figures 10 and 12), with an OR less than 1, suggesting that sarcoidosis might exert a protective effect by reducing vitamin B12 levels.

However, in this reverse MR analysis, the association strengths between pneumoconiosis as an exposure and calcium levels and vitamin E levels as outcomes were relatively low, with ORs of 1.003 and 0.999, respectively, suggesting that the potential impacts between them are not significant. These findings indicate that CRDs have significant impacts on specific micronutrient levels, providing new scientific evidence, but further research is necessary to elucidate the roles of these nutrients in disease prevention and treatment. According to Table 2, vitamins and minerals, in the MR-RAPS analysis method for CRDs, OR > 1 indicates that the increase of the mineral level may increase the risk of CRDs; OR < 1 indicates that it may have a protective effect. It is further confirmed that when vitamin B12 is increased, the risk of sarcoidosis will increase, while the risk of pneumoconiosis will increase with vitamin B12 and decrease with the increase of calcium level.

## 4. Discussion

This study reveals the potential causal relationships between specific micronutrients and CRDs through the MR analysis. The findings indicate a bidirectional causal relationship between calcium and asthma, whereas the causal relationship between pneumoconiosis and calcium levels, although positive, is of lower magnitude. Among the vitamins, vitamin B12 shows a risk causal relationship with asthma, while sarcoidosis is associated with reduced vitamin B12 levels. Vitamin E exhibits a protective causal relationship with IPF, and pneumoconiosis shows a protective relationship with vitamin E, though the association is less pronounced. These results suggest the significant role of specific micronutrients in the prevention and management of CRDs.

### 4.1. Analysis of Causal Relationship Between Calcium and CRDs

Calcium plays a critical role in maintaining the normal function of the immune system, particularly in regulating inflammatory responses and promoting immune cell functions [22]. Calcium is essential for the signaling of various immune cells such as T cells, B cells, and macrophages and is involved in cell activation, proliferation, and cytokine secretion [23]. Adequate calcium levels help maintain normal T cell function, prevent excessive inflammatory responses, and play a crucial role in the production and secretion of antibodies by B cells [24]. Calcium also regulates the functions of macrophages and other inflammatory cells, reducing airway inflammation and inhibiting the release of inflammatory cytokines like interleukin-6 (IL-6) and tumor necrosis factor-*α* (TNF-*α*), effectively lowering airway inflammation [25, 26]. Additionally, calcium is involved in mast cell degranulation, stabilizing the mast cell membrane and reducing the release of allergic mediators such as histamine, thereby mitigating allergic reactions [27].

On the other hand, asthma, a chronic inflammatory disease, significantly affects the health of millions of people worldwide. The pathogenesis of asthma involves complex genetic and environmental factors, with inflammatory mediators playing a crucial role [28]. Asthma patients exhibit elevated levels of inflammatory mediators (e.g., interleukins, tumor necrosis factor), which can interfere with calcium metabolism and distribution. Chronic inflammation may lead to the release of calcium from bones into the bloodstream, resulting in elevated blood calcium levels and decreased tissue calcium levels [29, 30]. Furthermore, medications commonly used in asthma treatment, such as glucocorticoids, can affect calcium absorption and excretion [31]. The long-term use of glucocorticoids may reduce calcium absorption and increase its excretion, leading to calcium deficiency [32]. Glucocorticoids are frequently used anti-inflammatory drugs in asthma management, but their long-term use not only affects calcium metabolism but can also cause complications like osteoporosis. Asthma patients often present with metabolic syndrome, where metabolic disorders can disrupt calcium regulatory mechanisms, further affecting calcium levels [33]. Metabolic syndrome includes obesity, insulin resistance, hypertension, and dyslipidemia, which collectively exacerbate the condition of asthma patients and influence calcium balance through complex metabolic pathways [34]. Therefore, a bidirectional causal relationship may exist between calcium and asthma: calcium reduces asthma risk by regulating immune function and reducing inflammation, while asthma influences calcium levels through inflammatory mediators, medication treatment, and metabolic disorders [35, 36]. These factors suggest that asthma might alter calcium metabolism through multiple mechanisms, including medication side effects, inflammation, and metabolic disturbances. Thus, the potential for reverse causality between asthma and calcium is significant and warrants further exploration in future studies.

Previous studies have revealed the pathological mechanisms between calcium ions and asthma [37, 38]. For example, the expression of calcium-sensing receptor (CaSR) is significantly increased in asthma patients, and its antagonist NPS2143 can alleviate asthma symptoms by reducing airway hyperresponsiveness (AHR) and inflammation [39, 40]. This suggests that calcium ions play a crucial role in the pathogenesis of asthma, and their intervention can significantly improve asthma symptoms. Based on the above pathological mechanisms, this study verifies the bidirectional causal relationship between calcium ions and asthma through MR, suggesting that increasing calcium intake can reduce asthma risk. Additionally, Luo et al. found through MR analysis that higher levels of magnesium and vitamin D are associated with increased CA risk [41]. However, this study did not support this finding, possibly due to the different instrumental variables included in the analysis. In the clinical application, targeted dietary adjustments can be made according to different patients, such as asthma patients treated with glucocorticoid for a long time, with dosage individualized based on factors like age, calcium levels, and comorbid conditions. Additionally, the careful monitoring of calcium intake is recommended for patients with cardiovascular diseases or allergies. Managing calcium metabolism could improve prognosis and reduce inflammation in asthma patients, but further clinical trials are needed to establish optimal supplementation levels. Additionally, if vitamin B12 is identified as a risk factor for asthma, clinical recommendations may need to focus on carefully monitoring the vitamin B12 supplementation. Excessive intake of vitamin B12 could potentially exacerbate asthma symptoms, and supplementation should be considered on a case-by-case basis, taking into account the patient's overall health status and nutrient balance.

This study also found a potential risk causal relationship between pneumoconiosis and calcium ion levels. Currently, the etiology of pneumoconiosis is clear: it is a lung disease caused by prolonged exposure to mineral dust, primarily inorganic particles including silica, coal dust, and asbestos fibers [42]. The development of pneumoconiosis is often accompanied by chronic inflammation, which not only directly leads to lung tissue damage and functional loss but also affects systemic metabolism through complex mechanisms [43, 44]. Although MR analysis results show a causal relationship between pneumoconiosis and calcium ions, considering the low magnitude of the association (OR = 1.003, 95% CI 1.002–1.004), this study suggests that the negative causal effect of pneumoconiosis on calcium ions requires further verification.

### 4.2. Analysis of Causal Relationship Between Vitamin B12 and CRDs

Vitamin B12 plays a crucial role in cellular metabolism and DNA synthesis, and it also has significant effects on the immune system. In epigenetic studies of asthma, DNA methylation plays an important role in the occurrence and development of asthma [45]. DNA methylation regulates gene expression by adding methyl groups to DNA molecules, and abnormal methylation levels of various genes in the airways and immune cells of asthma patients have been observed [46, 47]. B vitamins, particularly folic acid, vitamin B6, and vitamin B12, are involved in human DNA methylation [48]. Folic acid acts as a methyl donor in a one-carbon metabolism, and its active form, 5-methyltetrahydrofolate, is involved in converting homocysteine to methionine, a process requiring vitamin B12 as a coenzyme [49]. Vitamin B6 plays a role in transamination and deamination, providing the necessary conditions for a one-carbon unit metabolism. A deficiency in B vitamins can lead to elevated homocysteine levels, affecting the efficiency and accuracy of DNA methylation, potentially resulting in abnormal gene expression and disease occurrence [50]. Additionally, vitamin B12 is crucial for the function of T cells and B cells; T cells are responsible for identifying and attacking infected or damaged cells, while B cells produce antibodies to combat foreign pathogens [51].

Comparing our findings with those of other researchers in the same field, we can observe some similarities and differences. For instance, a cross-sectional study by Zhang et al. found that the vitamin B2 intake increased the risk of asthma [52]. Since vitamin B2 and vitamin B12 belong to the B vitamin group, it is worth considering whether their mechanisms in asthma pathogenesis overlap. However, a study by Subramaniam et al. found lower vitamin B12 levels in children with poorly controlled asthma [53]. In contrast, our study identified a negative causal effect between vitamin B12 and asthma, differing from Subramaniam et al.'s findings. Therefore, the causal relationship between vitamin B12 and asthma requires further validation. The differences might stem from study design, statistical methods, and potential confounders and interactions. Future research should use standardized experimental conditions and sample handling methods and apply uniform statistical analysis techniques to ensure the comparability and reliability of the results. Such comparisons and interpretations will allow for a more accurate assessment of the role of vitamin B12 in the pathogenesis of asthma, providing scientific evidence for the development of effective prevention and treatment strategies.

Our study also found a potential protective causal relationship between sarcoidosis and vitamin B12 levels. Sarcoidosis is a multisystem inflammatory disease characterized by noncaseating granulomas in various organs, commonly affecting the lungs, lymph nodes, skin, and eyes [54]. Current research on the relationship between sarcoidosis and vitamins has mostly focused on vitamin D. Sarcoidosis patients often exhibit abnormal vitamin D metabolism, primarily due to the overexpression of 1-*α*-hydroxylase in granulomas, leading to an increased conversion of 25-hydroxyvitamin D to the active form 1,25-dihydroxyvitamin D [55, 56]. Elevated levels of 1,25-dihydroxyvitamin D can result in hypercalcemia, a common complication of sarcoidosis [56, 57]. Research has indicated that vitamin D supplementation in sarcoidosis patients should be approached with caution, as it may exacerbate the risk of hypercalcemia [58]. In contrast to the existing research, our study did not find potential causal relationships between sarcoidosis and other vitamins, including vitamin D. Research on the relationship between sarcoidosis and vitamin B12 is limited. In a case report by Vronsky et al., a patient with Lofgren's syndrome exhibited migratory arthritis, erythema nodosum, and bilateral hilar lymphadenopathy, along with severe vitamin B12 deficiency. The patient showed improvement in symptoms and a significant increase in vitamin B12 levels after treatment with prednisone and vitamin B12 supplementation. However, vitamin B12 deficiency reappeared when steroid therapy was reduced, suggesting that vitamin B12 deficiency may be related to sarcoidosis [59]. Our study's findings suggest a potential protective causal effect of sarcoidosis on vitamin B12 levels, but this needs further validation. Future research should explore the specific role and mechanisms of vitamin B12 in the development and treatment of sarcoidosis, providing more scientific evidence for clinical management.

### 4.3. Analysis of Causal Relationship Between Vitamin E and CRDs

IPF is a fatal chronic lung disease characterized by progressive lung fibrosis, leading to the gradual deterioration of respiratory function. IPF patients often exhibit high levels of oxidative stress, which accelerates lung tissue damage and fibrosis [60]. Oxidative stress not only causes cellular structure damage but also triggers a series of inflammatory responses, worsening the disease [61]. Vitamin E, a potent antioxidant, has anti-inflammatory and anti-fibrotic properties. It can neutralize free radicals at the cellular and tissue levels, reducing oxidative stress-induced lung tissue damage [62, 63]. Vitamin E may stabilize cell membranes, prevent lipid peroxidation, and protect cells from apoptosis induced by oxidative stress [64]. Chronic inflammation plays a central role in the pathogenesis of IPF, with increased infiltration and activity of inflammatory cells damaging alveolar structures and promoting fibrosis progression [65]. Vitamin E can reduce the intensity of inflammatory responses by inhibiting the production of proinflammatory factors such as TNF-*α* and IL-6 [66].

Although the occurrence of IPF is often associated with low levels of vitamin D, most research on the relationship between vitamins and IPF has focused primarily on vitamin D, with fewer studies exploring the role of vitamin E in this context. For example, Chang et al. demonstrated in an IPF mouse model that vitamin D can inhibit lung fibrosis [67]. However, Lin et al. [68] and our research found that while vitamin D levels are associated with IPF, no causal relationship was established between vitamin D and IPF in MR research. These findings suggest that the relationship between vitamin D and IPF is more likely to be associative rather than causal. The lack of causality in both studies may stem from limitations inherent in MR, such as horizontal pleiotropy or unmeasured confounding factors. It is important to note that while vitamin E shows potential protective effects on IPF, its causal relationship with IPF remains inconclusive. Future studies with larger and more diverse samples are needed to further explore the potential role of vitamin D and other vitamins in IPF.

Regarding the protective effect of pneumoconiosis on vitamin E, our study found a significant result, but the association strength was low (OR = 0.999, 95% CI 0.999–1.000). Thus, the positive causal effect of pneumoconiosis on vitamin E requires further validation.

### 4.4. Study Limitations and Future Directions

This study used MR methods to reveal causal relationships between certain micronutrients and CRDs, but several limitations must be acknowledged. Although MR methods effectively reduce confounding bias present in traditional observational studies, enhancing causal inference credibility, the genetic instrumental variables used have weak associations with exposures. This may lead to insufficient statistical power, making the detected causal relationships less robust. Another important limitation is the potential confounding from asthma medications, such as glucocorticoids, which can affect calcium metabolism and may influence the observed relationship between calcium and asthma, a factor not fully controlled for in this study. Additionally, limitations such as small sample sizes, unidentified confounders, complex gene–environment interactions, temporal factors, and measurement errors could affect the results. The instrumental variables used in this study were derived from European populations, limiting the generalizability of the results. Furthermore, while we used MR-Egger and MR-PRESSO to account for pleiotropy, there remains a possibility of horizontal pleiotropy, where calcium SNPs may influence respiratory diseases via non-nutritional pathways, such as immune modulation or bone-related effects. These alternative pathways were not fully explored and could contribute to the associations observed.

Future studies should focus on selecting genetic variations with stronger associations to exposures, increasing sample sizes, and incorporating environmental factors to better understand the causal relationships. Additionally, exploring alternative pathways and conducting long-term follow-up studies with high-precision measurement tools will enhance the accuracy and reliability of the findings. By refining study designs and considering gene–environment interactions, future research can provide stronger evidence, ultimately supporting the development of more effective prevention and treatment strategies for CRDs.

## 5. Conclusion

This study explored the causal relationships between 12 micronutrients and common CRDs using bidirectional two-sample MR methods. The results indicate significant causal associations among calcium, vitamin B12, and vitamin E with CRDs. Specifically, there is a bidirectional protective causal relationship between calcium and asthma, suggesting that increasing calcium intake may reduce the risk of asthma. However, the causal relationships among other vitamins, minerals, and CRDs remain inconclusive, necessitating further research to validate these findings' robustness and generalizability.

## Figures and Tables

**Table 1:** Detailed information on micronutrient and vitamin data.

GWAS ID	Exposure	Sample size	Number of SNPs	Population
ebi-a-GCST90025990	Calcium	400,792	4,218,949	European
ieu-a-1073	Copper	2603	2,543,646	European
ukb-b-20447	Iron	64,979	9,851,867	European
ukb-b-7372	Magnesium	64,979	9,851,867	European
ieu-a-1075	Selenium	2874	2,451,527	European
ieu-a-1079	Zinc	2603	2,543,610	European
ukb-b-19524	Vitamin B12	64,979	9,851,867	European
ukb-b-7864	Vitamin B6	64,979	9,851,867	European
ukb-b-15175	Vitamin C	460,351	9,851,867	European
ukb-b-9596	Vitamin A	460,351	9,851,867	European
ukb-b-12648	Vitamin D	460,351	9,851,867	European
ukb-b-12506	Vitamin E	460,351	9,851,867	European

**Table 2:** The analysis results by MR-RAPS method.

Exposure	Outcome	OR	OR_lci95	OR_uci95	*p* value
Vitamin A	Asthma	0.993	0.758	1.3	0.958
	COPD	0.405	0.003	49.193	0.712
	IPF	0.992	0.946	1.041	0.753
	Pneumoconiosis	70,175.832	0	2.79669*E* + 16	0.413
	Sarcoidosis	0.113	0	214,314.004	0.767

Vitamin B12	Asthma	1.015	0.997	1.033	0.098
	COPD	1.154	0.831	1.603	0.392
	IPF	1	0.997	1.003	0.881
	Pneumoconiosis	4.505	0.764	26.557	0.0963
	Sarcoidosis	1.039	0.433	2.493	0.932

Vitamin B6	Asthma	1.007	0.995	1.019	0.234
	COPD	1.016	0.818	1.263	0.884
	IPF	1.001	0.998	1.003	0.576
	Pneumoconiosis	0.916	0.273	3.073	0.887
	Sarcoidosis	1.361	0.731	2.533	0.331

Vitamin C	Asthma	1.597	1.463	1.742	< 0.001
	COPD	0.319	0.057	1.784	0.193
	IPF	1.007	0.99	1.024	0.413
	Pneumoconiosis	0.993	0	14,940.94	0.999
	Sarcoidosis	0.996	0.007	145.515	0.999

Vitamin D	COPD	0.568	0.019	16.923	0.744
	IPF	1.007	0.974	1.041	0.699
	Pneumoconiosis	9916.178	0	1.97663*E* + 12	0.345
	Sarcoidosis	0.002	0	71.526	0.252

Vitamin E	Asthma	1.066	0.864	1.315	0.55
	COPD	1.153	0.026	51.75	0.942
	IPF	0.95	0.915	0.986	0.00771
	Pneumoconiosis	0.175	0	256690503.9	0.871
	Sarcoidosis	0.062	0	3389.473	0.617

Calcium	Asthma	0.995	0.991	0.998	0.00538
	COPD	NA	NA	NA	NA
	IPF	1	1	1.001	0.166
	Pneumoconiosis	0.938	0.64	1.376	0.745
	Sarcoidosis	1.256	1.03	1.533	0.0244

Copper	Asthma	0.998	0.995	1	0.0923
	COPD	1.019	0.984	1.055	0.288
	IPF	1	0.999	1.001	0.952
	Pneumoconiosis	1.045	0.849	1.286	0.678
	Sarcoidosis	1.046	0.944	1.159	0.393

Iron	Asthma	1.012	0.997	1.027	0.123
	COPD	0.824	0.632	1.074	0.152
	IPF	0.999	0.997	1.002	0.639
	Pneumoconiosis	0.795	0.189	3.339	0.754
	Sarcoidosis	0.836	0.4	1.749	0.635

Magnesium	Asthma	0.998	0.986	1.011	0.806
	COPD	0.922	0.738	1.151	0.472
	IPF	0.999	0.997	1.002	0.623
	Pneumoconiosis	0.458	0.137	1.524	0.203
	Sarcoidosis	1.173	0.649	2.121	0.596

Selenium	Asthma	1.001	0.997	1.004	0.677
	COPD	1.022	0.963	1.086	0.468
	IPF	1	1	1.001	0.567
	Pneumoconiosis	0.936	0.675	1.299	0.694
	Sarcoidosis	1.02	0.859	1.212	0.818

Zinc	Asthma	0.998	0.995	1.001	0.251
	COPD	1.012	0.961	1.065	0.657
	IPF	1	1	1.001	0.54
	Pneumoconiosis	1.11	0.831	1.483	0.481
	Sarcoidosis	1.072	0.919	1.25	0.379

## Data Availability

The data for this study were sourced from the IEU GWAS database (https://gwas.mrcieu.ac.uk/). We collected data on calcium, copper, iron, magnesium, selenium, zinc, vitamin B12, vitamin B6, vitamin C, vitamin A, vitamin D, and vitamin E.

## Supporting Information

Supporting Information 1 Supporting Table 1: Sensitivity analysis of mineral micronutrients and chronic respiratory diseases' heterogeneity and pleiotropy results.

Supporting Information 2 Supporting Table 2: Sensitivity analysis of vitamin micronutrients and chronic respiratory diseases' heterogeneity and pleiotropy results.
